# Platelet-derived major histocompatibility complex class I coating on *Treponema pallidum* attenuates natural killer cell lethality

**DOI:** 10.1080/21505594.2024.2350892

**Published:** 2024-05-14

**Authors:** Qiu-Yan Xu, Xin-Qi Zheng, Wei-Ming Ye, Dong-Yu Yi, Ze Li, Qing-Qi Meng, Man-Li Tong, Dan Liu, Tian-Ci Yang

**Affiliations:** a Centre of Clinical Laboratory, Zhongshan Hospital of Xiamen University, School of Medicine, Xiamen University, Xiamen, China; b Institute of Infectious Disease, School of Medicine, Xiamen University, Xiamen, China

**Keywords:** Immunosurveillance, natural killer cells, MHC class I, platelets, Treponema pallidum

## Abstract

The evasive tactics of *Treponema pallidum* pose a major challenge in combating and eradicating syphilis. Natural killer (NK) cells mediate important effector functions in the control of pathogenic infection, preferentially eliminating targets with low or no expression of major histocompatibility complex (MHC) class I. To clarify *T. pallidum’s* mechanisms in evading NK-mediated immunosurveillance, experiments were performed to explore the cross-talk relations among *T. pallidum*, NK cells, and platelets. *T. pallidum* adhered to, activated, and promoted particle secretion of platelets. After preincubation with *T. pallidum*, platelets expressed and secreted high levels of MHC class I, subsequently transferring them to the surface of *T. pallidum*, potentially inducing an immune phenotype characterized by the “pseudo-expression” of MHC class I on the surface of *T. pallidum* (hereafter referred to a “pseudo-expression” of MHC class I). The *polA* mRNA assay showed that platelet-preincubated *T. pallidum* group exhibited a significantly higher copy number of *polA* transcript than the *T. pallidum* group. The survival rate of *T. pallidum* mirrored that of *polA* mRNA, indicating that preincubation of *T. pallidum* with platelets attenuated NK cell lethality. Platelets pseudo-expressed the MHC class I ligand on the *T. pallidum* surface, facilitating binding to killer cell immunoglobulin-like receptors with two immunoglobulin domains and long cytoplasmic tail 3 (KIR2DL3) on NK cells and initiating dephosphorylation of Vav1 and phosphorylation of Crk, ultimately attenuating NK cell lethality. Our findings elucidate the mechanism by which platelets transfer MHC class I to the *T. pallidum* surface to evade NK cell immune clearance.

## Introduction

Syphilis, a sexually and vertically transmitted infection caused by the spirochaete *Treponema pallidum* subspecies *pallidum* [1], has seen a resurgence in global incidence rates [2]. This persistence can be attributed in part to the ability of *T. pallidum* to escape host immune clearance mechanisms [3], which is a major challenge for the control and eradication of syphilis. The mechanism through which *T*. *pallidum* escapes host clearance can be viewed as a conflict between the invasive capacity of *T. pallidum* and the proficiency of host immune responses in eliminating spirochaetes [4]. Natural killer (NK) cells are indispensable constituents of the innate immune system form the frontline defence against tumours and pathogens [5]. Despite this, only a few studies have investigated the role of NK cells in *T. pallidum* infection [6–8]. A previous study showed a substantial infiltration of NK cells in *T. pallidum* infected skin, in addition to CD4^+^ and CD8^+^ T cells. Conversely, the number of NK cells in the peripheral blood of patients with secondary syphilis was markedly reduced and accompanied by the presence of immature NK cell subtypes [7]. NK cell function is intricately regulated by a delicate balance of signals transduced through activating and inhibitory receptors [9]. Typically, the triggering of NK cell activation is initiated through two primary modes: “induced-self” recognition and “missing-self” recognition [10]. The “induced-self” recognition mechanism necessitates the interaction between stress-induced or virus-encoded ligands on target cells and germline-encoded activating receptors. Conversely, the “missing-self” hypothesis posits the selective elimination of target cells exhibiting low or absent major histocompatibility complex (MHC) class I expression by NK cells. This elimination process is hindered by the binding of MHC class I molecules present on target cells to inhibitory receptors known as killer cell immunoglobulin-like receptors (KIRs) gene family [11]. Among these receptors, KIR2DL3 is an inhibitory member that shares a common preference for human leukocyte antigen I binding, specifically from the C1 group [12]. Interestingly, the VAPWNSFAL peptide, while not inherently inhibitory to KIR2DL3-positive NK cells, can antagonize the inhibition caused by a peptide strongly inhibiting NK cells, rather than being functionally neutral [13].

Several studies have shown that platelets act as safeguards for circulating tumour cells against NK-mediated immunosurveillance [14–16]. Platelets are among the first to sense, decorate, or react to pathogens in circulation [17] and play a crucial role in the infection process of *T. pallidum*, which primarily occurs through bloodstream transmission [18]. With further exploration of platelet function, various studies have focused on the involvement of platelets in the pathogenesis of invasive bacteria such as *Staphylococcus aureus*, *Streptococcus pyogenes*, and *Borrelia species* [19–21]. *Borrelia* were reported to be highly concentrated in the platelet fraction of the blood of a patient with Lyme disease, indicating the potential for platelets to serve as a reservoir for *Borrelia* [22]. Our previous study showed that *T. pallidum* Tp0136 promotes platelet activation [23], and Cameron et al. reported that *T*. *pallidum* is able to activate platelets [18]. However, despite these findings, it remains uncertain whether platelets serve as reservoirs for *T*. *pallidum* as in the case of *Borrelia*, thereby aiding *T*. *pallidum* in evading host immune clearance.

Given these findings, it is worth investigating whether the interaction between *T*. *pallidum* and platelets can promote the pseudo-expression of platelet-derived MHC class I on the surface of *T*. *pallidum* (hereafter referred to as “pseudo-expression” of MHC class I) and trigger NK cells to generate the “missing-self,” thereby attenuating the response of NK cells to *T*. *pallidum*. Herein, we aimed to explore the adhesion and activation of *T. pallidum* to platelets and investigate the transfer of MHC class I by platelets in the vicinity of *T. pallidum*, which confer pseudo-expression of MHC class I on *T. pallidum*, thus interfering with the recognition and killing of NK cells. We provide mechanistic insights into how *T*. *pallidum* escapes the immune clearance of NK cells, which is crucial for unravelling the immune escape strategy of *T*. *pallidum*.

## Materials and methods

### Preparation of NK cells and platelets

The NK cell line NK92 presents an appealing alternative to donor NK cells, as it can be cultured *in vitro* and has demonstrated safety and efficacy in clinical trials [24–26]. NK92 cells were purchased from Procell Culture Collection (Procell Life Science & Technology Inc., Wuhan, China) and cultured in a special supporting medium supplemented with 100 U of recombinant interleukin (IL)-2. Platelets were prepared as previously described [23] and diluted to 2.0 × 10^8^/mL with modified Tyrode’s solution (Solarbio, Beijing, China) for subsequent experiments.

#### *T. pallidum* propagation and extraction

The *T. pallidum* Nichols strain was provided by Prof. Lorenzo Giacani (University of Washington, Seattle, WA, USA). The propagation and harvesting of *T. pallidum* were performed as described by Li et al. [27] under microaerophilic conditions of approximately 5 % oxygen in a Coy Laboratory Products Anaerobic Chamber (Mandel Scientific Company Inc., Guelph, ON, Canada) to enhance *T. pallidum* viability.

## Detection of the co-localization between *T. pallidum* and platelets

To detect *T. pallidum* and platelet co-localization, flow cytometry was performed according to a slight adaptation of a previously described method [18]. *T. pallidum* (1 × 10^7^/mL, 50 μL) and platelets (2.0 × 10^8^/mL, 200 μL) were mixed and cultured in a microaerophilic chamber, and stained with an APC-labelled anti-human CD41 antibody (Biolegend, Shanghai, China) as a platelet identifier and with an FITC-labelled anti-*T. pallidum* antibody (Abcam, Cambridge, MA, USA) prior to analysis using flow cytometry (BD FACSCanto II, NJ, USA). The data were analysed using FlowJo software (TreeStar Software, Ashland, OR, USA).

## Observation of *T. pallidum* adhesion to platelets using microscopy

The adhesion of *T*. *pallidum* to platelets was monitored using dark-field microscopy, scanning electron microscopy, and confocal immunofluorescence microscopy. For dark-field microscopy, *T. pallidum* (1 × 10^7^/mL, 50 μL) and platelets (2.0 × 10^8^/mL, 200 μL) were mixed for co-culture. The *T*. *pallidum*-platelet mixture sample volume was limited to 2 μL per 0.13–0.16 mm thick glass slide with an 18 × 18 mm cover glass gently pressed into place, then viewed using a Nikon Eclipse 80i dark-field microscope using a Nikon DS-Qi1Mc digital camera with NIS-Elements imaging software (Nikon Canada Inc., Mississauga, ON, Canada).

For scanning electron microscopy, *T*. *pallidum*-platelet mixture samples were fixed with 2.5 % glutaraldehyde overnight at 4°C, washed with 0.1 M phosphate buffer (pH 7.4), dehydrated in a graded ethanol series, freeze-dried, and gold-sputtered. The prepared samples were observed using a JSM-6390LV scanning electron microscope (SEM, S-4800, Hitachi Ltd., Tokyo, Japan).

For confocal immunofluorescence microscopy, *T*. *pallidum*-platelet mixture samples were placed onto fibrinogen-coated Millicell glass slides for 1 h at 37°C. Adherent mixtures were fixed with 4 % paraformaldehyde, blocked with 5 % bovine serum albumin, and stained with rabbit anti-*T. pallidum* antibodies at 4°C overnight, followed by incubation with a secondary antibody (fluorescein isothiocyanate-conjugated goat anti-rabbit IgG [Abcam, Cambridge, UK]) and Phalloidin-iFluor 555 Reagent (Abcam, Cambridge, UK) for 30 min at room temperature. Fluorescent images were obtained using a confocal microscope (Zeiss Axio Observer LSM780, Oberkochen, Germany).

## Platelet activation assays and particle secretion detection

Measurement of the platelet surface secretions, granule P-selectin, platelet factor 4 (PF4), and beta-thromboglobulin (β-TG), as indices of platelet activation were performed. For P-selectin expression in platelets, the *T. pallidum*-platelet mixture was analysed using flow cytometry, as previously reported [18]. The levels of PF4 and β-TG in the mixed samples were assessed using a Human PF4 Simple Step enzyme-linked immunosorbent assay (ELISA)® Kit (Abcam, MA, USA) and a Human β-TG ELISA Kit (Elabscience, Wuhan, China), respectively. The levels of platelet-derived growth factor (PDGF) and endothelial growth factor (EGF) released from the platelets into the supernatant were measured using a commercially available ELISA Kit (Jianglai Industrial Limited by Share Ltd., Shanghai, China).

## Analysis of MHC class I expression

The MHC class I secretion from the supernatant of the *T. pallidum*-platelet mixture was assessed using an ELISA kit (Proteintech Group, Illinois, USA). In addition, the distribution of MHC class I molecules in the platelets was detected using an immunofluorescence assay, in brief as follows: *T*. *pallidum*-platelet mixture samples were placed onto fibrinogen-coated Millicell glass slides for 1 h at 37°C. Adherent mixtures were fixed with 4 % paraformaldehyde, permeabilized with 0.1 % Triton X-100, blocked with 5 % bovine serum albumin, and then stained with rabbit anti-MHC class I antibody (Abcam, Cambridge, USA) at 4°C overnight, followed by incubation with a secondary antibody for 30 min at room temperature. Fluorescence images were obtained using a confocal microscope.

Furthermore, immunoelectron microscopy was used to analyse MHC class I expression in *T. pallidum* as previously described [28]. The *T. pallidum*-platelet mixture was fixed with 4 % paraformaldehyde and 0.2 % glutaraldehyde for 24 h and washed twice with 0.1 M phosphate buffer (pH 7.0). Samples were incubated with anti-MHC class I antibody and 15 nm Goat Anti-Rabbit IgG/Gold (Solarbio, Beijing, China), stained with freshly filtered 2 % phosphotungstic acid, and examined under an FEI Tecnai G2 Spirit transmission electron microscope (FEI, Hillsboro, Oregon, USA) at an accelerating voltage of 120 kV.

## Detection of the *polA* transcript and survival rate of *T. pallidum*

*T. pallidum* was cultured with either platelets or the VAPWNSFAL peptide (Sangon Biotech Co., Ltd., Shanghai, China), a potent inhibitor of NK cells known to induce signalling inhibition [29], and then co-cultured with NK cells. DNA was extracted from the cells using a QIAamp DNA Mini Kit (QIAGEN INC., Valencia, CA, USA) following the manufacturer’s instructions. Furthermore, total RNA was extracted using an RNA Simple Total RNA Kit (TANGEN Biotech Co., Ltd., Beijing, China), treated with DNase I (Invitrogen, Carlsbad, CA) to remove DNA, and was then reverse transcribed with a Reverse Transcription Kit (TransGen Biotech, Beijing, China). The *T*. *pallidum polA* transcript that specifically detects *T. pallidum* DNA representing the total load of *T*. *pallidum* [30] was amplified with a 20 μL reaction as previously reported [31] and monitored using a Roche Cobas Z 480 system (F. Hoffmann-La, Roche, Ltd., Basel, Switzerland). The primers used to target *polA* were 5-TACGGTGCAAGTGCTCAGAC-3 (sense) and 5-CAGGCACATTGTCGGAGGAA-3 (antisense). The *T*. *pallidum* survival rate percentage was calculated using the following formula: *polA* mRNA copies/*polA* DNA copy number × 100.

## Detection of inhibitory receptor-associated signals

*T. pallidum* was cultured with platelets (or VAPWNSFAL peptide) for 6 h respectively, then co-cultured with NK cells, and immunoprecipitation experiments were performed as previously reported [29]. Antibodies including anti-phosphor-Vav1 (Y174), anti-Vav1, anti-phospho-Crk (Y221), anti-Crk, anti-killer cell immunoglobulin-like receptors two Ig domains and long cytoplasmic tail 3 (KIR2DL3), and β-actin were purchased from Abcam (Cambridge, USA). Total cellular proteins were subjected to SDS-PAGE and analysed using western blotting [32]. ImageJ software (ImageJ Fiji, NIH, USA) was used to analyse the band intensities, while data analysis was performed using GraphPad Prism 7 (GraphPad Software, North Parker, USA) and IBM SPSS statistics version 26 (SPSS, Inc., Chicago, IL, USA) software.

## Statistical analysis

Data are presented as mean ± standard deviation (SD) of three independent experiments. Statistical analysis was performed using GraphPad Prism 7 (GraphPad Software, North Parker, USA) and IBM SPSS statistics version 26 (SPSS, Inc., Chicago, IL, USA) software. An unpaired Student’s *t*-test was used to compare two groups, and one-way analysis of variance (ANOVA) to compare multiple groups. *p* < 0.05 was considered as statistically significant.

## Results

### T. pallidum adhered to platelets

**Figure 1.**
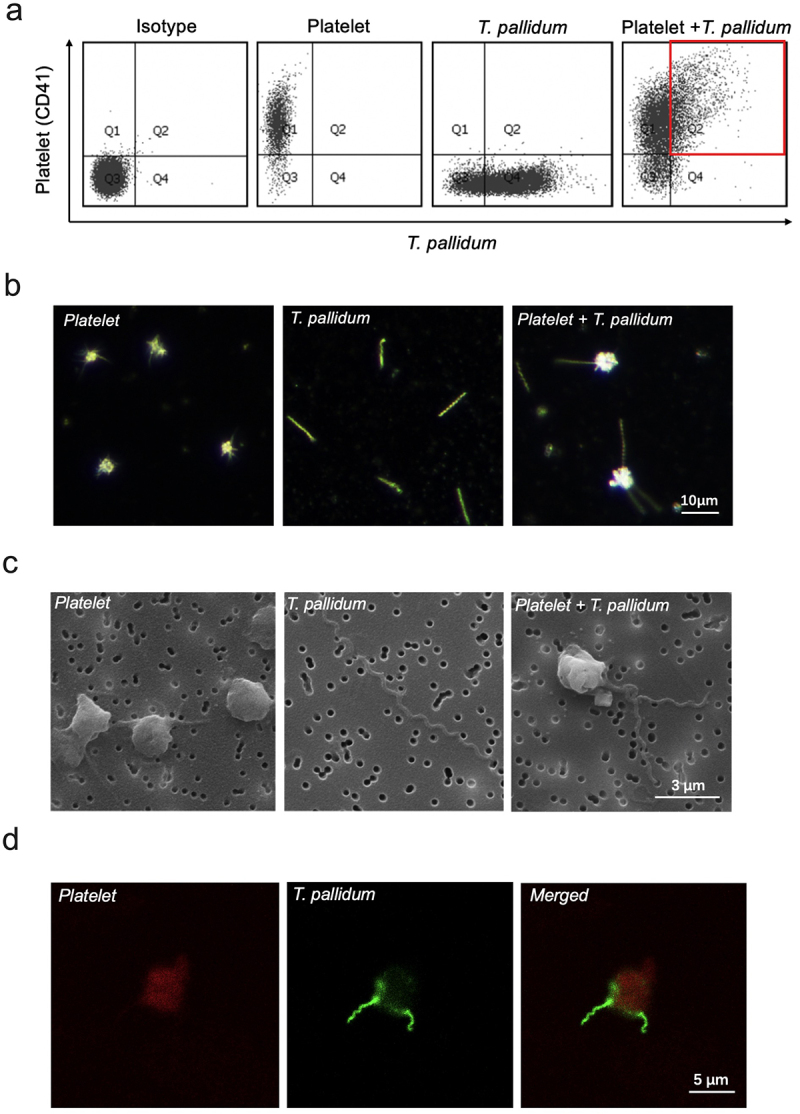
*Treponema pallidum* adhered to the platelets. (a)The co-localization of platelets and *T*. *pallidum* was detected using flow cytometry. (b-d)*T*. *pallidum*-platelet attachment events were monitored using dark-field (b), scanning electron (c), and confocal immunofluorescence microscopy (d).

To determine the co-localization of *T*. *pallidum* and platelets, *T*. *pallidum* and platelets were co-cultured in a microaerobic chamber. Flow cytometric analysis showed that *T*. *pallidum* and platelets co-localized, forming a binding event (red area in Figure 1a). Furthermore, to investigate the interaction between *T*. *pallidum* and platelets, the attachment of *T*. *pallidum* to platelets was monitored using three kinds of microscopy: dark-field (Figure 1b), scanning electron (Figure 1c), and confocal immunofluorescence (Figure 1d). Microscopic results showed that *T*. *pallidum* adhered to platelets, effectively forming a physical shield.

#### T. pallidum activated platelets and promoted platelet-secreting particles

**Figure 2.**
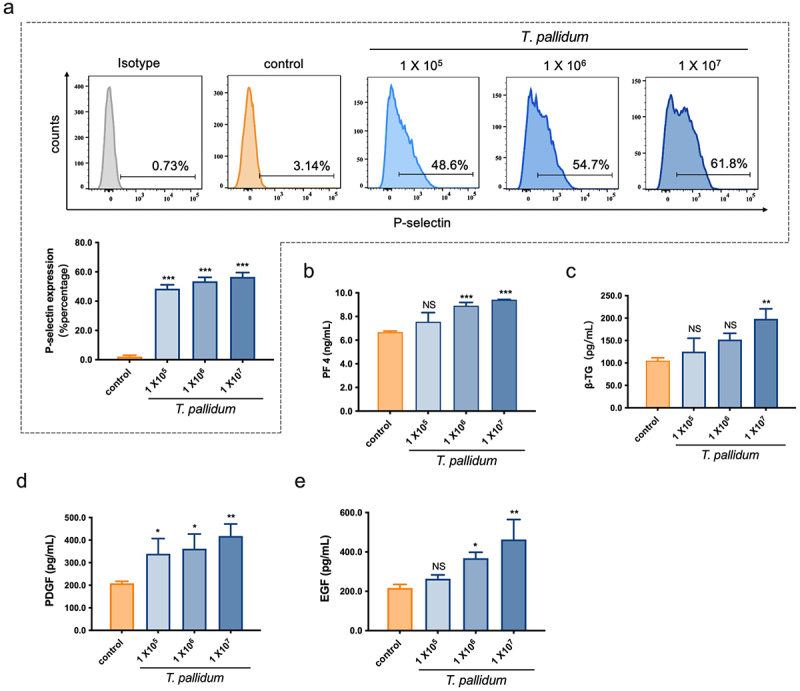
*T*. *pallidum* activated platelets and promoted platelet-secreting particles. (a)Activated platelets with *T. pallidum* were assessed using flow cytometry. (b-e)*T*. *pallidum*-induced platelets secretion of platelet factor 4 (PF4) (b), beta-thromboglobulin (β-TG) (c), platelet-derived growth factor (PDGF) (d), and endothelial growth factor (EGF) (e) was analyzed using enzyme-linked immunosorbent assay (ELISA). These data represent differentiation experiments performed across three independent experiments containing three duplicates. Data are represented as means ± standard deviation (SD) (*n* = 3). One-way analysis of variance (ANOVA) was used to compare multiple groups. NS, not significant; *p < 0.05; **p < 0.01; ***p < 0.001.

The ability of *T. pallidum* to activate platelets was investigated through quantifying platelet P-selectin expression using flow cytometry. Flow cytometry analysis showed that co-incubation of *T. pallidum* (physiological saline as a control) with platelets elicited a significant increase in P-selectin expression in a concentration-dependent manner (*p <* 0.001) (Figure 2a). Platelet activation by *T. pallidum* was also assessed through detecting platelet secretion particles. The ELISA results showed that the secretion of PF4 was significantly increased when the incubation concentration of *T*. *pallidum* was 1 × 10^6^/mL (*p <* 0.001) (Figure 2b), and the platelet secretion of β-TG increased significantly when incubated with 1 × 10^7^/mL *T*. *pallidum* (*p <* 0.01) (Figure 2c).

In addition, changes in PDGF and EGF secretion levels were measured to further evaluate platelet secretion in response to *T. pallidum* using ELISA. The results showed that co-incubation of *T. pallidum* with platelets elicited a significant increase in PDGF secretion in a concentration-dependent manner (*p* < 0.05) (Figure 2d), and EGF secretion by platelets also increased significantly when the incubation concentration of *T*. *pallidum* was > 1 × 10^6^/mL (*p* < 0.05) (Figure 2e). These results indicate that *T*. *pallidum* activates platelets and promotes the platelet secretion of particles such as PF4, β-TG, PDGF, and EGF.

## Pseudo-expression of MHC class I on platelet-preincubated *T. pallidum*

**Figure 3.**
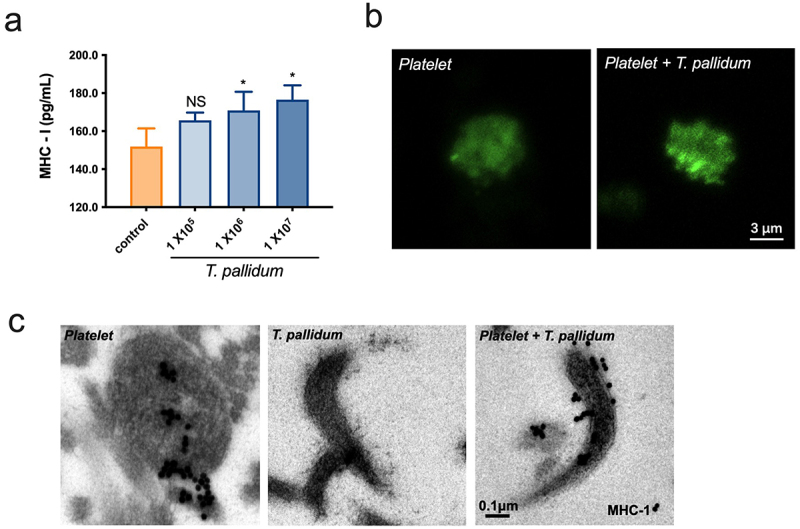
Pseudo-expression of major histocompatibility complex (MHC) class I on platelet-preincubated *T. pallidum*. (a) The secretion of MHC class I using ELISA assay. These data represent differentiation experiments performed across three independent experiments. Data are represented as means ± SD (*n* = 3). One-way ANOVA was used to compare multiple groups. NS, not significant; *p < 0.05. (b) The distribution of MHC class I was analyzed using confocal microscopy. (c) Analysis of MHC class I expression with post-embedding immunogold labelling (black dots) using electron microscopy.

Platelets express a large amount of MHC class I molecules, which play a central role in immune surveillance by presenting antigens to lymphocytes [33]. ELISA results revealed that the secretion of MHC class I in *T. pallidum* activated platelets significantly increased at an incubation concentration of 1 × 10^6^/mL with *T*. *pallidum* (*p* < 0.05) (Figure 3a). The confocal microscopy results further showed that MHC class I (green fluorescence) reached the plasma membrane, presented an edge effect in the platelet-preincubated *T. pallidum* group, and displayed a tendency towards degranulation (Figure 3b). Electron microscopy was used to investigate the ultrastructures. Immunogold staining for MHC class I revealed that platelet-derived MHC class I enveloped the periphery of *T. pallidum* (Figure 3c). Taken together, these data demonstrate that platelets preincubated with *T. pallidum* expressed high levels of MHC class I, and transferred them to *T. pallidum* surface, which may lead to conferring an immune phenotype of “pseudo-expression” of MHC class I on *T. pallidum*.

## Preincubation of *T. pallidum* with platelets attenuated NK cell lethality

**Figure 4.**
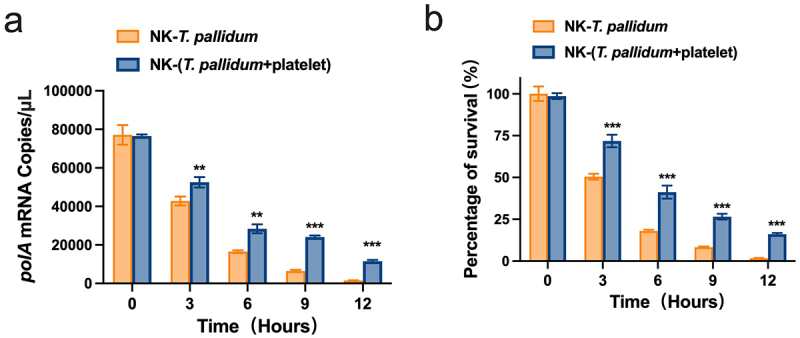
Preincubation of *T. pallidum* with platelets attenuated natural killer (NK) cell lethality. (a) Analysis of *T. pallidum* mRNA through targeting the *polA* transcript. (b) Survival rate analysis of *T. pallidum*. These data represent differentiation experiments performed across three independent experiments. Data are represented as means ± SD (*n* = 3). A student’s *t*-test was used to compare two groups. **p < 0.01; ***p < 0.001.

Given the above results, we evaluated the efficacy of NK cells in eliminating *T. pallidum* by monitoring fluctuations in the mRNA transcription levels of *T. pallidum polA*. The lower the copy number of the *T. pallidum polA* transcript, the lower the vitality of *T. pallidum*. The *polA* mRNA assay showed that after co-culture with NK cells for 3 h, the platelet-preincubated *T. pallidum* group exhibited a significantly lower copy number of the *polA* transcript than the *T. pallidum* group (*p* < 0.01). When the co-culture time was extended to 12 h, the difference between the two groups became more pronounced (*p* < 0.001) (Figure 4a). The survival rate of *T. pallidum* was similar to that of *polA* (*p* < 0.001) (Figure 4b), indicating that preincubation of *T. pallidum* with platelets attenuated NK cell lethality.

## Preincubation of *T. pallidum* with platelets attenuated NK cell lethality via pseudo-expression of platelet-derived MHC class I

**Figure 5.**
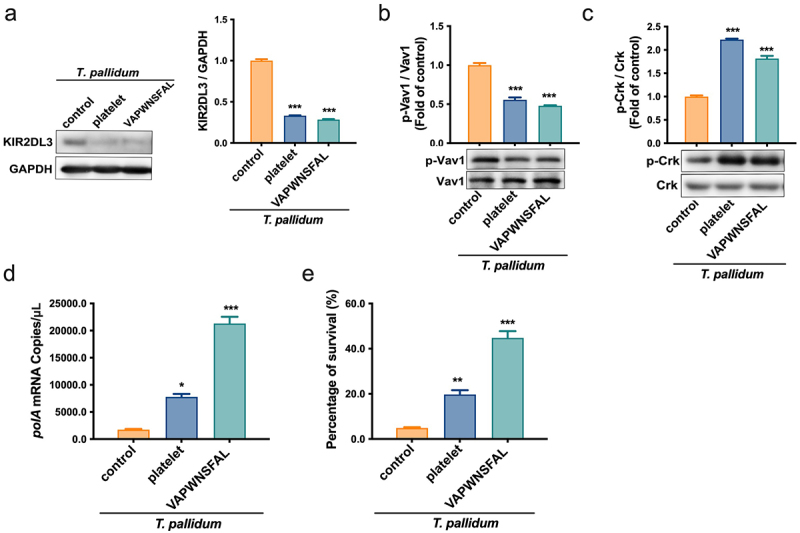
*T. pallidum* with platelets attenuated NK cell lethality via pseudo-expression of platelet-derived MHC class I. (a) Cell lysates were immunoprecipitated to analyze killer cell immunoglobulin-like receptors two immunoglobulin domains and long cytoplasmic tail 3(KIR2DL3). (b) Analysis of cell lysates for Vav1 signaling using western blotting. (c) Analysis of cell lysates for Crk signaling using western blotting. (d) Analysis of *T. pallidum* mRNA through targeting the *polA* transcript. (e) Survival rate analysis of *T. pallidum*. These data represent differentiation experiments performed across three independent experiments. Data are represented as means ± SD (*n* = 3). One-way ANOVA was used to compare multiple groups. NS, not significant; *p < 0.05; **p < 0.01; ***p < 0.001.

The immune response of NK cells is controlled by a balance of signals transduced by activating and inhibitory receptors [34]. To further study the inhibitory effect of MHC class I pseudo-expression on *T. pallidum* in NK cell responsiveness, the recruitment of the inhibitory receptor KIR2DL3 to NK cells, which have MHC class I ligands [35], was detected using a co-immunoprecipitation assay. The results showed that the recruitment level of KIR2DL3 in the platelet-preincubated *T. pallidum* group was significantly lower than that in the control group (*p* < 0.001) (Figure 5a). This indicates that most of the unrecruited KIR2DL3 receptors in the platelet-preincubated *T. pallidum* group may bind to MHC and trigger inhibition signals in NK cells. Simultaneously, the immunoprecipitation assay also showed that the recruitment level of KIR2DL3 in the VAPWNSFAL peptide-preincubated *T. pallidum* group was similar to the platelet-preincubated *T. pallidum* group (*p* < 0.001) (Figure 5a). To further investigate whether the inhibitory signal transduction machinery of NK cells induced by MHC class I pseudo-expression in *T. pallidum* was involved in this process, the KIR2DL3-associated signal was detected. The protein expression results showed that *T. pallidum* preincubated with platelets or VAPWNSFAL peptide alone induced dephosphorylation of Vav1 in NK cells (*p* < 0.001) (Figure 5b). During inhibition, the small adaptor protein Crk showed the opposite result and promoted phosphorylation (*p* < 0.001) (Figure 5c). The *polA* mRNA experiment showed that platelet (or VAPWNSFAL peptide) preincubation attenuated the killing effect of NK cells on *T. pallidum* in contrast to the control group (Figure 5d), which was also confirmed using the survival test of *T. pallidum* (Figure 5e). These results suggest that platelets pseudo-express MHC class I ligands to *T. pallidum*, bind to KIR2DL3 receptors of NK cells, and initiate dephosphorylation of Vav1 and Crk, thereby inhibiting the recognition and killing of *T. pallidum* by NK cells.

## Discussion

Strategies that enable *T. pallidum*, the aetiologic agent of venereal syphilis, to evade host defence are poorly understood. Several invasive bacteria have been shown to exploit normal platelet function during infection. NK cells play an important role in the initial defence against various pathogens, including viruses and intracellular bacteria [36]. In this study, we provided *in vitro* evidence that *T*. *pallidum* adheres to platelets, activates them, and promotes their particle secretion, effectively forming a physical shield and immune barrier. Additionally, platelets assign pseudo-expression of MHC class I ligands to the *T. pallidum* surface, enabling their binding to the KIR2DL3 receptor on NK cells, initiating the dephosphorylation of Vav1 and phosphorylation of Crk, and ultimately attenuating NK cell lethality.

Platelets are dynamic effector cells that cross the immune and inflammatory continuum. In 1968, Gasic et al. identified the effect of platelets on metastasis through reducing the number of lung metastases after tail vein injection of tumour cells in mice [37]. Palumbo et al. found that platelet activation, which is related to the adhesion of tumour cells and release of platelet particles, is crucial for their metastasis-facilitating effects [14]. Our results showed that *T*. *pallidum* adheres to platelets, which may be the basis for the blood transmission by *T*. *pallidum* via platelets. Additionally, a kinetic study reported that *T. pallidum* can activate platelets and preferentially interact with them [18]. These results were corroborated by the results of this study, which showed that *T*. *pallidum* activated platelets and promoted the particle secretion of platelets such as PF4, P-selectin, β-TG, PDGF, and EGF. These secretory factors and particles wrap around *T. pallidum*, forming an immune barrier that interferes with host immune cell surveillance.

MHC class I molecules are found on the surfaces of nucleated cells and platelets [38]. Our data suggest that granules containing MHC class I molecules are transferred to the platelet membrane and released after *T. pallidum* activated platelets. Platelet-derived MHC class I wraps around *T. pallidum* and induces enhanced MHC class I expression on the *T. pallidum* surface, effectively providing a decoy cover that allows for immune escape. NK cells are cytotoxic lymphocytes that are components of innate immunity [39] and play an essential role in controlling infections caused by certain viruses. NK cell reactivity is guided by recognition of “missing-self” and “induced-self,” implying that NK cells kill target cells or pathogenic bacteria with a low or absent expression of MHC class I molecules (“missing-self”) [40]. The “missing-self” hypothesis implies that this mechanism of NK activity is of special interest in pathogenic infections. This also supports our finding that *T. pallidum* evades immune surveillance by NK cells through the pseudo-expression of MHC class I.

**Figure 6.**
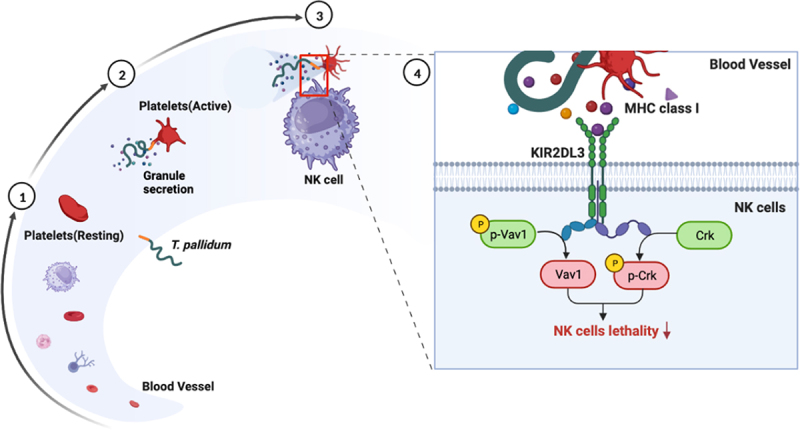
Schematic illustration of mechanisms underlying platelet-derived MHC class I on *T. pallidum* attenuated NK cell lethality. (1) *T. pallidum* invaded and spread through the blood. (2) *T. pallidum* activated platelets and promoted platelet secretion particles, while also pseudo-expressing platelet-derived MHC class I in the *T. pallidum* surface. (3) Pseudo-expression of platelet-derived MHC class I on *T. pallidum* escaped from the immune clearance of NK cells. (4) Mechanism explanation: platelet-derived MHC class I coating on *T. pallidum* binds to KIR2DL3 receptors of NK cells, initiates dephosphorylation of Vav1 and phosphorylation of Crk changes, and then attenuates NK cell lethality.

Reactions between NK cell receptors and MHCs allow for considerable possibilities for the regulation of NK cell function. The control of NK cell reactivity by inhibitory receptors specific to MHC class I is well established [41]. The most frequently described mechanism is the relationship between KIR and MHC ligands. One report showed that recipients who carried KIR2DL3 more often developed active cytomegalovirus infections [42], which is consistent with our study showing that the KIR2DL3 inhibitory receptor plays an important role in the lethal response of NK cells in relation to co-incubation of *T. pallidum* with platelets. The MHC class I-specific inhibitory receptor KIR2DL3 signals through its cytosolic immunoreceptor tyrosine-based inhibition motifs in their cytoplasmic tail to recruit tyrosine phosphatase SHP‐1 upon ligand engagement [43,44]. This recruitment leads to dominant inhibitory signals that dephosphorylate the guanine nucleotide exchange factor, Vav1. Dephosphorylation of Vav1 inhibits NK cell activity and prevents downstream signalling events associated with NK cell activation [45]. During KIR2DL3-mediated inhibition, the small adaptor protein Crk is phosphorylated. Phosphorylated Crk can further modulate downstream signalling pathways, contributing to the overall inhibitory effect of KIR2DL3 [46]. Our data suggest that platelets assign pseudo-expression of MHC class I ligands to *T. pallidum*, which bind to the KIR2DL3 receptors of NK cells, initiate dephosphorylation of Vav1 and phosphorylation of Crk, and inhibit the recognition and killing of *T. pallidum* by NK cells (Figure 6). This was supported by related experiments on the VAPWNSFAL peptide, which were also consistent with previous reports [29].

To the best of our knowledge, this is the first *in vitro* study showing that a platelet-derived MHC class I coating provides *T. pallidum* with a physical shield against NK cells. However, this study had several limitations. First, we only used an NK cell line, which may not accurately mimic the behaviour of primary NK cells and CD8^+^ T immune cells in the body. Second, there are individual differences in the KIRs gene phenotype, and employing platelets and NK cells derived from the same individual would enhance the credibility of the findings. A breakthrough in the technology for overexpressing or silencing KIR genotypes on NK92 cells, along with examining different NK cell allogeneic responses and donor-recipient KIR genotypes, could provide further validation for our study. Third, using high concentrations of *T. pallidum* in this study, as described by Church et al. [18], may not accurately reflect the concentration of *T. pallidum* in the early stage of syphilis infection in humans. Therefore, it is imperative to replicate the real scenarios *in vivo* through further experiments. Additionally, most data presented are *in vitro*, owing to the limited techniques for genetic engineering and animal modelling of *T. pallidum*, coupled with ethical limitations in that the associated disease should not be fully reproduced in individuals. More direct and powerful evidence from *in vivo* experiments or in individuals is highly desirable. Additional genotype analyses of KIR and human leukocyte antigens could be considered for sequencing or *in vitro* experiments involving patients with syphilis and healthy individuals. Previous studies have reported an association between the KIR1D/KIR1D genotype and susceptibility to syphilis in the Chinese Han population, which belongs to KIR gene haplotype A [8].

In summary, the mechanism of immune escape of *T. pallidum* was confirmed to be through physically cloaking the *T. pallidum* surface with MHC class I molecules produced by platelets, effectively conferring an immune phenotype of MHC class I pseudo-expression to *T. pallidum*. Therefore, *T. pallidum* evades “missing-self” recognition, thereby undermining NK cell immunosurveillance. Through leveraging platelets, *T. pallidum* not only overcomes immune surveillance, but also manipulates the host immune response, enabling survival and progression within the host. This study provides novel insights into the mechanisms employed by *T. pallidum* to evade host immune clearance, which are crucial for elucidating its immune escape strategy in relation to *T*. *pallidum*.

## Author contribution

T.-C. Y., D. L., and Q.-Y. X. conceived and designed the project. Q.-Y. X., X.-Q. Z., and W.-M. Y. performed laboratory experiments. M.-L. T., D.-Y. Y., Z. L., and Q.-Q M. analysed and interpreted the data. Q.-Y. X. and T.-C. Y. wrote the paper with input from all other authors. M.-L. T., D. L., and T.-C. Y. supervised the project.

## Data Availability

The authors confirm that the data supporting the findings of this study are available within the article and/or its supplementary materials.
